# Extended Stability Evaluation of Selected Cathinones

**DOI:** 10.3389/fchem.2020.597726

**Published:** 2020-11-13

**Authors:** Heather L. Ciallella, Lexus R. Rutter, Lorna A. Nisbet, Karen S. Scott

**Affiliations:** ^1^Forensic Science, Department of Chemistry and Physics, Arcadia University, Glenside, PA, United States; ^2^Forensic and Investigative Science, School of Life Science, Anglia Ruskin University, Cambridge, United Kingdom

**Keywords:** stability, cathinone, GC/MS (gas chromatograph/mass spectrometry), solvents, blood, urine

## Abstract

Understanding the stability of drugs in a forensic toxicology setting is critical for the evaluation of drug concentrations. Synthetic cathinones are new psychoactive substances structurally derived from cathinone, the psychoactive component of *Catha edulis* (“khat”), a shrub that is indigenous to the Middle East and East Africa. Previous research has evaluated the stability of synthetic cathinones in biological matrices, including blood preserved with the combination of NaF and K_2_C_2_O_4_ used in gray-top tubes. However, it does not assess their stability in blood preserved with Na_2_EDTA, used for some clinical samples. Further, stability in unpreserved urine samples was only studied for two weeks. This research evaluates the stabilities of four Schedule I synthetic cathinones: mephedrone, MDPV (3,4-methylenedioxypyrovalerone), naphyrone, and α-PVP (alpha-pyrrolidinopentiophenone) at 20°C (room temperature), 4°C (refrigerator), and −20°C (freezer). Stability was assessed in methanolic and acetonitrile solutions, as well as in Na_2_EDTA-preserved blood and unpreserved urine. Solutions (1 mg/L) of each drug in each matrix stored in aliquots (100 μL, solvents; 1.2 mL, biological samples; *n* = 12) at each of the three temperatures for triplicate analysis on days 3, 7, 14, and 30. On day 0 of each study, three additional aliquots of each solution were analyzed. Biological samples underwent solid-phase extraction before analysis. All samples were analyzed in full-scan by gas chromatography-mass spectrometry (GC-MS). The results of this study show that under room temperature and refrigerator storage conditions, mephedrone, naphyrone, and MDPV will degrade in methanol. This degradation starts are early as day 3. Additionally, all four drugs will degrade in Na_2_EDTA-preserved human whole blood samples in at least one evaluated storage environment. However, when in acetonitrile-based working solutions and unpreserved urine samples, they proved to be more stable. Methanolic working solutions and samples of Na_2_EDTA-preserved blood containing these cathinones should be stored in the freezer and used or tested with urgency to ensure that quantitative sample analysis is as accurate as possible in forensic casework.

## Introduction

**Figure 1:**
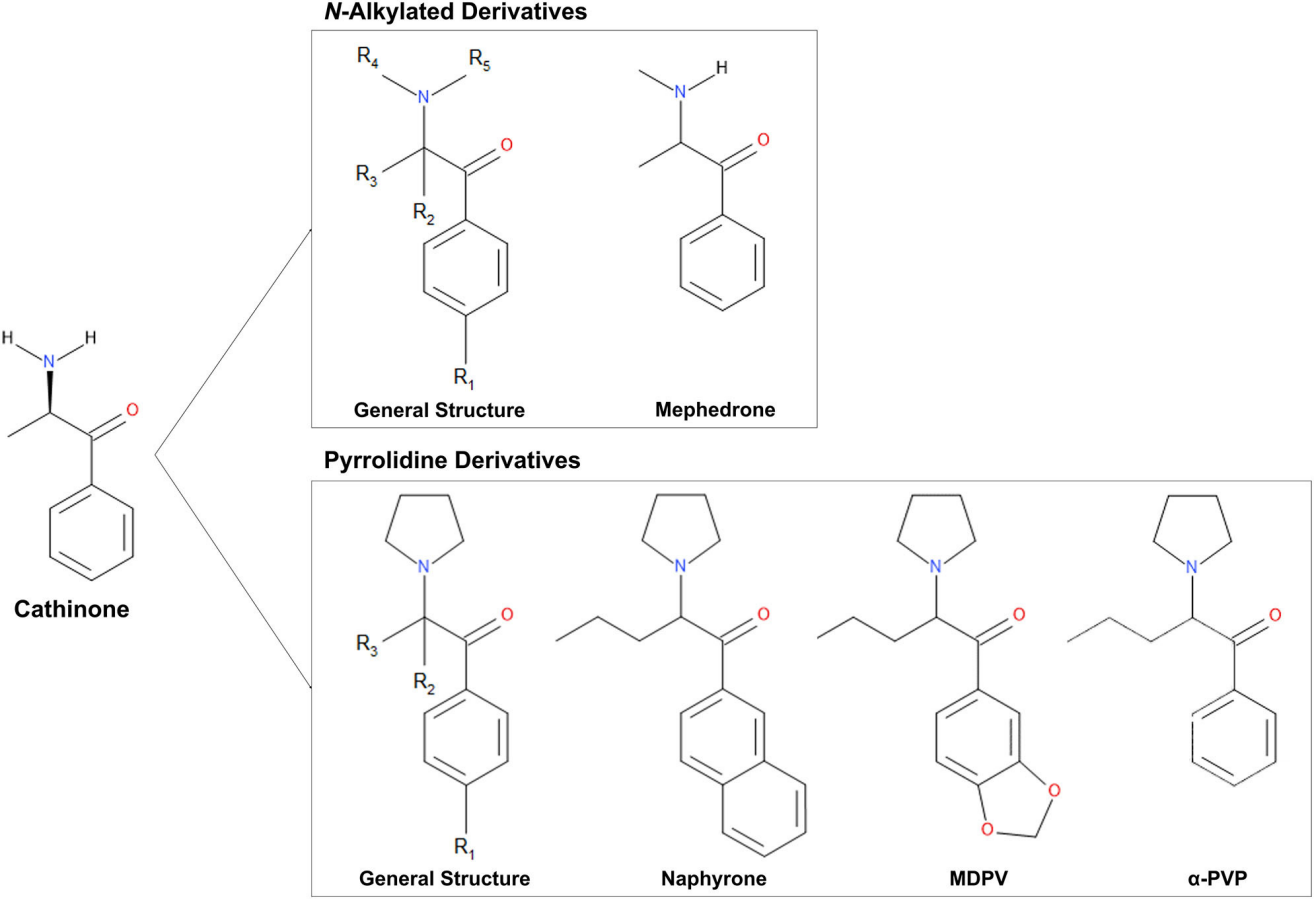
Chemical structures of cathinone and N-alkylated derivatives and pyrrolidine derivatives evaluated in this study.

Cathinone (Figure 1) is the naturally occurring psychoactive component of *Catha edulis* (“Khat”), a plant that is indigenous to the Middle East and East Africa (Halbach, 1972; Krikorian, 1984). The structure of cathinone is similar to that of amphetamines. However, it contains an additional carbonyl group at the β position, which decreases its ability to cross the blood-brain barrier and, therefore, its potency (Gibbons and Zloh, 2010). As the structural similarities suggest, cathinone induces similar effects to amphetamines (Zelger et al., 1980; Kalix and Khan, 1984; Brenneisen et al., 1990). Cathinone is a mixed-acting sympathomimetic central nervous system stimulant (Halbach, 1972; Kalix and Khan, 1984). Its main effects likely result from increased bioavailability of certain neurotransmitters and include euphoria, increased alertness, hypertension, and tachycardia (Kalix and Khan, 1984; Bentur et al., 2008; Patel, 2009; Simmler et al., 2013).

The United Nations defines new psychoactive substances (NPS) as “substances of abuse, either in a pure form or a preparation, that are not controlled by the 1961 Single Convention on Narcotic Drugs or the 1971 Convention on Psychotropic Substances, but which may pose a public health threat” (United Nations Office on Drugs Crime, 2013). Standard colloquial terms for these drugs include “designer drugs” and “legal highs,” as they are sold online or in head shops, and their primary purpose is to circumvent international drug legislation (United Nations Office on Drugs Crime, 2013). This category of drugs includes a broad range of substances, including phenethylamines and piperazines (Rosenbaum et al., 2012). Synthetic cathinone derivatives are among the most widely used new psychoactive substances (5). Although most NPS are newly available on the illicit drug market, many of these compounds were synthesized many years ago, such as the synthetic cathinone MDPV (3,4-methylenedioxypyrovalerone), for which the original synthesis dates back to 1969 (Yohannan and Bozenko, 2010).

Synthetic cathinones (Figure 1) are new psychoactive substances and structural derivatives of cathinone (Rosenbaum et al., 2012; Abdullah et al., 2014; Banks et al., 2014; Katz et al., 2014; Pieprzyca et al., 2020). The class of synthetic cathinones encompasses a large number of compounds because the cathinone molecule has several places at which the addition and modification of functional groups are possible. Synthetic cathinones can either be *N-*alkylated or have a pyrrolidine ring connecting R groups 4 and 5 (Figure 1) (Katz et al., 2014). Synthetic cathinones pose a significant public health risk, mainly due to their inconsistent purity (Davies et al., 2010) and frequent co-administration with other drugs (Katz et al., 2014).

Head shops and Internet retailers sell synthetic cathinones under names such as “bath salts” and “plant food” and are often labeled “not for human consumption” to circumvent international legislation (Rosenbaum et al., 2012; Ross et al., 2012; Shanks et al., 2012; United Nations Office on Drugs Crime, 2013; Hohmann et al., 2014). In 2010, synthetic cathinone use spiked worldwide, likely due to a decrease in purity of cocaine and 3,4-methylenedioxymethamphetamine (MDMA), which rendered concerns about the relatively lower potency of synthetic cathinones less important to consumers (Measham et al., 2010; Prosser and Nelson, 2012). Unlike other commonly abused drugs such as methamphetamine, which were approved for therapeutic use and went through full clinical trials, synthetic cathinones were identified as dangerous before the completion of extensive clinical trials (Prosser and Nelson, 2012; Hohmann et al., 2014; Papaseit et al., 2017). Therefore, the reliable toxicological information available in the scientific literature regarding these compounds is limited (Prosser and Nelson, 2012; Rosenbaum et al., 2012). Like cathinone, synthetic cathinone users report effects that closely mimic the effects of amphetamines (Prosser and Nelson, 2012). Their hypothesized mechanism of action is a promotion of the release of neurotransmitters (dopamine, serotonin, and norepinephrine) with dose-dependent effects (Cozzi et al., 1999; Prosser and Nelson, 2012; Simmler et al., 2013). However, the use of these drugs appears to trigger additional adverse effects, namely delusions, hallucinations, and reckless behavior (Kasick et al., 2012; Murray et al., 2012; Prosser and Nelson, 2012).

Sunlight and heat are known to degrade the cathinone in *Catha edulis* into dimers or inactive metabolites (Chappell and Lee, 2010; Katz et al., 2014). Stability research on cathinone's synthetic derivatives tells a similar story, with many synthetic cathinones showing some degree of instability based on storage temperature and sample matrix (Sørensen, 2011; Tsujikawa et al., 2012; Al-Saffar et al., 2013; Concheiro et al., 2013; Johnson and Botch-Jones, 2013; Maskell et al., 2013; Soh and Elliott, 2014; Busardò et al., 2015; Glicksberg and Kerrigan, 2017, 2018; Miller et al., 2017; da Cunha et al., 2018; Glicksberg et al., 2018; Adamowicz and Malczyk, 2019; Czerwinska et al., 2019; Nowak et al., 2020). Understanding the consequences of this variability on subsequent interpretations of data derived from samples suspected to contain synthetic cathinones is crucial. Room temperature stability is often variable for synthetic cathinones, which does not pose a threat to most laboratories, as it is standard procedure to store samples in the refrigerator or freezer. However, room temperature instability does have a substantial impact on post-mortem toxicology, where the time between death and sample collection at room temperature could cause inaccurate results. Of additional concern is the conditions used to transport samples from the point of collection to the laboratory. These concerns about stability can become heightened if the drugs also exhibit variability in the refrigerator and freezer in both working solutions and biological matrices. The instability presented in refrigerator and freezer storage environments has an impact on the toxicological analysis of samples associated with post-mortem, driving under the influence (DUI), and drug-facilitated sexual assault (DFSA) casework.

This research aimed to determine the effects of storage temperature and matrix on the stability of four Schedule I synthetic cathinones (mephedrone, naphyrone, MDPV, and α-PVP). Currently, the most cited relevant study evaluates only one derivative, mephedrone, in unpreserved equine blood samples (Soh and Elliott, 2014). Previous synthetic cathinone stability studies primarily focus on blood samples preserved with the combination of sodium fluoride (NaF) and potassium oxalate (K_2_C_2_O_4_) in gray-top tubes. However, they do not assess their stability in blood preserved with Na_2_EDTA, used for some clinical samples, or in unpreserved human urine. Further, no previous research provides insight into the stability of these compounds in solvent-based working solutions. The results of this study provide a more comprehensive overview of the stability of these compounds in biological matrices over a more extended period, with an alternative preservative as well as the inclusion of solvent-based working solutions.

## Materials and Methods

### Chemicals and Reagents

Mephedrone, naphyrone, and mephedrone-D_3_ as 1 mg/mL solutions were purchased from Lipomed (Cambridge, Massachusetts, USA). Naphyrone-D_5_ 100 μg/mL solution was purchased from Cerilliant (Round Rock, Texas, USA). MDPV and α-PVP reference standards (5.0 mg) were purchased from Cayman Chemical Company (Ann Arbor, Michigan, USA) for the preparation of samples and calibrators. MDPV-D_8_ and α-PVP-D_8_ reference standards (100 μg/mL in methanol) were purchased from Cerilliant Corporation (Round Rock, Texas, USA). Methanol (MeOH), dichloromethane (DCM), ethyl acetate (EtOAc), ammonium hydroxide (NH_4_OH), isopropyl alcohol (IPA), and glacial acetic acid were all analytical grade and purchased from Pharmco by Greenfield Global (Brookfield, Connecticut, USA). Pentafluoropropionic anhydride (PFPA) was purchased from Sigma Aldrich (St Louis, MO, USA).

Blank human whole blood preserved with Na_2_EDTA was purchased from Golden West Biologicals (Temecula, California, USA). Blank human urine was self-collected under Arcadia University Institutional Review Board (IRB) proposal #17-01-16 and was tested unpreserved. Blank human whole blood and urine were screened for the presence of any standard prescription, over-the-counter, and recreational drugs before their use in this study. CLEAN SCREEN® CSDAU506 solid-phase extraction (SPE) cartridges with a combination of C_8_ (reverse phase) and benzenesulfonic acid (ion exchange) sorbent were purchased from United Chemical Technologies (UCT) (Bristol, Pennsylvania, USA).

### Calibration Curves

Calibrators were prepared by spiking 1 mL of the matrix of interest with a 10 mg/L drug stock solution and a 1 mg/L internal standard stock solution. Calibration curves were prepared by plotting peak area ratio (PAR) vs. the concentration of the associated calibrators. ChemStation Enhanced Data Analysis E.02.02.1431 calculated linear regression equations and correlation coefficients. SWGTOX guidelines require a correlation coefficient (*r*^2^) of 0.99 for each calibration curve and quantitated values within ±20% of their target values for suitable calibration models (Scientific Working Group for Forensic Toxicology, 2013).

### Sample Preparation and Analysis

**Figure 2:**
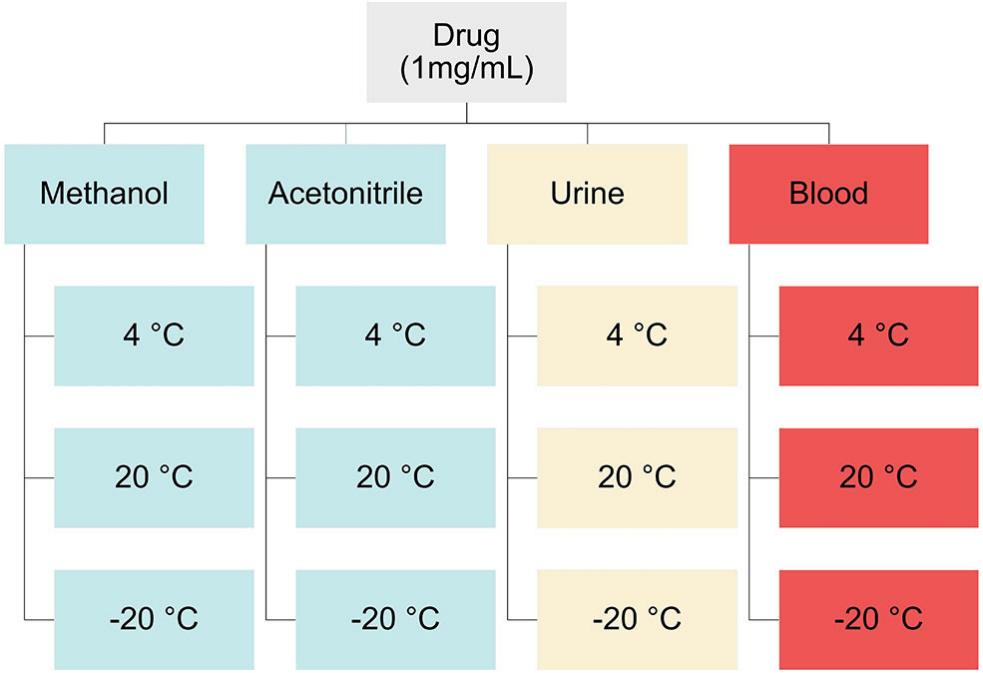
Experimental design and workflow used in this study.

Solvent samples for stability studies were prepared by pipetting 100 μL of 1 mg/L solutions of mephedrone, naphyrone, MDPV, or α-PVP into glass culture tubes (*n* = 48). Human whole blood and urine samples for stability studies were prepared in 60 mL of the biological matrix of interest to a concentration of 1 mg/L. These solutions were then aliquoted into Eppendorf® polypropylene microcentrifuge tubes (*n* = 48, 1.2 mL). Fifteen samples spiked with each drug in each matrix were stored at 20°C (room temperature), 4°C (refrigerator), and −20°C (freezer) temperatures (Figure 2). On days 0, 3, 7, 14, and 30, samples from each temperature were extracted in triplicate. Fifty microliters of the corresponding deuterated internal standard (mephedrone-D_3_ for mephedrone, naphyrone-D_5_ for naphyrone, MDPV-D_8_ for MDPV, α-PVP-D_8_ for α-PVP) was added to the solvent samples on the day of their extraction. Samples were then evaporated at 37°C under compressed air.

Before SPE for the blood and urine studies, 100 μL of the corresponding deuterated internal standard, 3 mL of 0.1 M pH 6 phosphate buffer, and 2 mL of dH_2_O were added to 1 mL of each sample and calibrator. The samples were then vortexed for 5 s and centrifuged for 10 min at 3,000 rotations per minute (rpm). SPE cartridges were conditioned with 3 mL MeOH, 3 mL dH_2_O, and 1 mL 0.1 M pH 6 phosphate buffer, and the supernatants from centrifuged samples were transferred into correspondingly labeled CLEAN SCREEN® CSDAU506 SPE cartridges. Cartridges were then washed with 3 mL dH_2_O, 1 mL 1 M acetic acid, and 3 mL MeOH and then dried under a vacuum pressure of at least 5 mmHg. Samples and calibrators were eluted with 3 mL of a 78:20:2 mixture of DCM/IPA/NH_4_OH, which was then evaporated at 37°C under compressed air. When using GC/MS to detect analytes, sample derivatization is often necessary before analysis. Derivatizing a compound changes a polar hydroxyl or amine group to a less polar functional group and increases the molecule's volatility, allowing for a better chromatogram. Derivatization often utilizes acylating agents. The polar N-H group of mephedrone was derivatized in this study using a 2:1 mixture of PFPA and EtOAc. After derivatization, the PFPA/EtOAc solution was evaporated to dryness. The dried down solutions for all four drugs were then reconstituted in 100 μg/mL of EtOAc and transferred to GC/MS vials for analysis.

### Instrumentation

Gas chromatography-mass spectrometry (GC-MS) analysis was carried out on an Agilent Technologies 7890 GC system/5973 EI-MS or Perkin Elmer Clarus® 680 GC system/SQ 8T MS. Equivalency between the two instruments was established based on calibration and controls. Once a stability study was started, the same instrument was used through the remainder of the 30 days. A Restek® Rtx®-5 fused silica column (30 m × 0.32 mm, 0.25 μm film thickness) was used for separation by GC. The MS was operated in full scan mode so that the method could be used for future stability studies in which scanning for degradation products would be required. The GC-MS method used for this analysis method was previously validated for the detection of 23 NPS, including synthetic cathinones (Nisbet et al., 2019). Although the previous study did not include α-PVP, the Nisbet et al. method was successfully validated for this analyte according to SWGTOX guidelines. Table 1 shows the retention times and ions used to monitor analyte stability throughout the study. Because the instrument was operated in full-scan, mass spectral matches were also made.

**Table 1:** Retention times and ions used for quantitation.

**Analyte**	**Retention time (min)**	**Quantitation ion (*m*/*z*)**	**Qualifier ion 1 (*m*/*z*)**	**Qualifier ion 2 (*m*/*z*)**
Mephedrone	9.43	160	204	323
Mephedrone-D_3_	9.40	163	207	326
Naphyrone	18.88	126	155	127
Naphyrone-D_5_	18.84	131	132	133
MDPV	17.98	126	149	110
MDPV-D_8_	17.93	134	133	135
α-PVP	12.59	126	124	127
α-PVP-D_8_	12.51	134	133	135

### Data Processing and Statistical Analysis

Peak area ratio and concentration were monitored to monitor the stability of the four drugs. Both peak area ratio (PAR) and linear regression are suitable methods for monitoring stability per SWGTOX validation guidelines (Scientific Working Group for Forensic Toxicology, 2013). At the appropriate retention times, peaks for each analyte and internal standard were present. Single-factor one-way analysis of variance (ANOVA) was performed on the data using Microsoft® Excel® Professional Plus 2013 to assess whether fluctuations in PAR or concentration were statistically significant throughout the 30-day studies. Performing an ANOVA evaluates whether variability in the data sets acquired in this analysis is attributed to differences in sample population means (μ_x_) or within-sample population variability (Peck et al., 2015). This statistic evaluated acquired data based on the null hypothesis (H_0_) that the mean PAR, or concentration, for days 0, 3, 7, 14, and 30 at a specific storage temperature are all equal to each other (Equation 1) (Peck et al., 2015). H_0_ is rejected for the alternative hypothesis (H_1_) that the mean PAR, or concentration, is significantly different for at least one sample population when the fluctuations in PAR or concentration cannot fully be explained by within-sample population variability (Peck et al., 2015). The ANOVA performed on data sets for this study assumed a significance level (α) of 0.05, meaning that there is a 5% chance that the null hypothesis is rejected despite being true (Peck et al., 2015). A test statistic (p) is calculated for the data, and if the *p*-value calculated for a data set falls below α, H_0_ is rejected. Values have been normalized as percent change from day 0 for ease of comparison among PARs in different matrices.

(1) $$\begin{array}{l} H_{0}:\mu_{Day\;0}=\mu_{Day\;3}=\mu_{Day\;7}=\mu_{Day\;14}=\mu_{Day\;30} \end{array}$$

where μ_*x*_ equals the sample PAR from day 0 to day *x*.

## Results and Discussion

### Calibration

**Figure 3:**
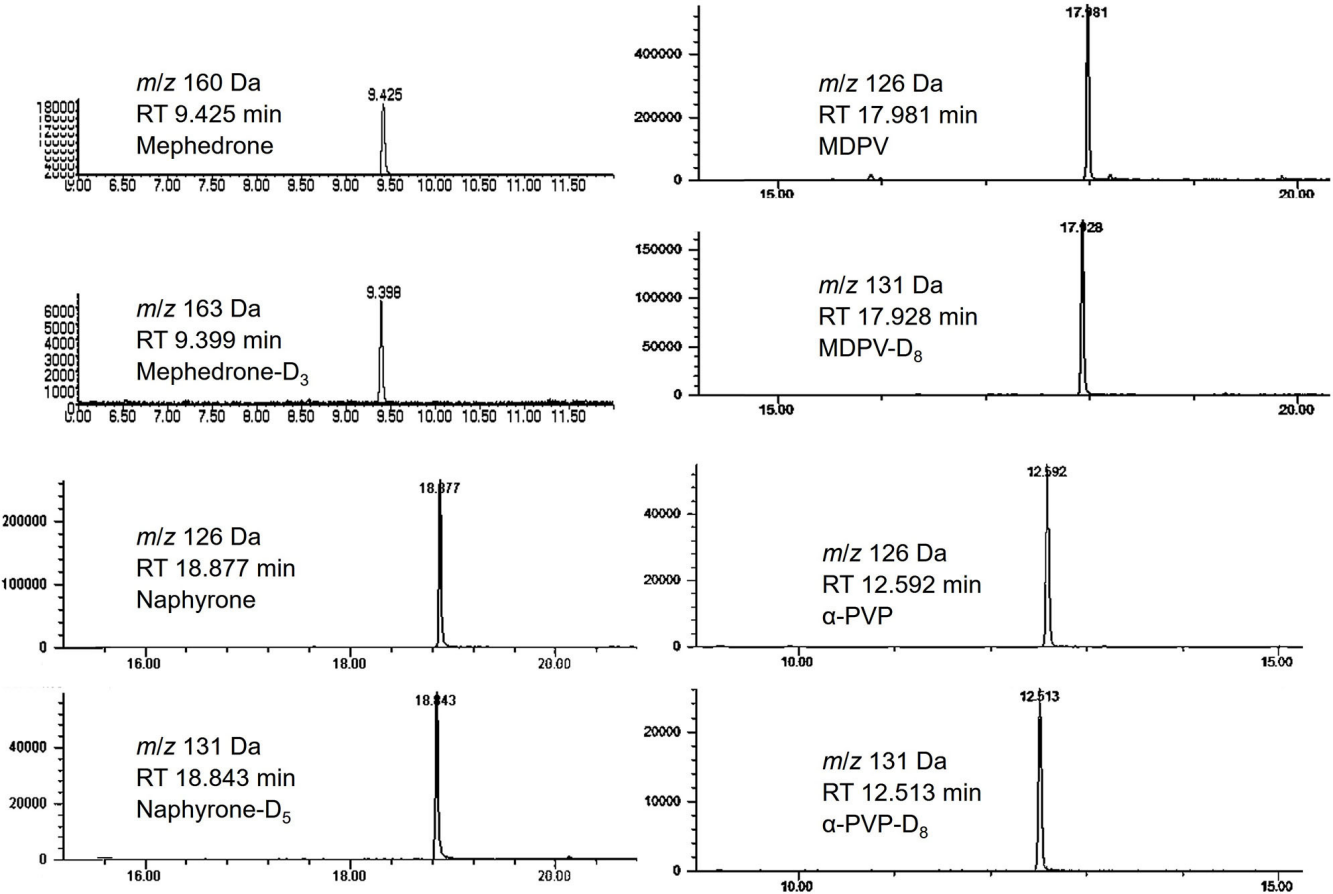
Chromatograms of mephedrone, MDPV, naphyrone, α-PVP and their respective internal standards.

Calibration curves were run prior to the analysis of each stability batch. All calibration curves produced acceptable *R*^2^ (>0.99) and consistent linear regression values across the expected concentration range. PAR was, therefore, acceptable to use for the determination of analyte loss during the studies. Extracted ion chromatograms for the quantification ion of each analyte and their respective internal standards are shown in Figure 3.

### Mephedrone Stability

**Figure 4:**
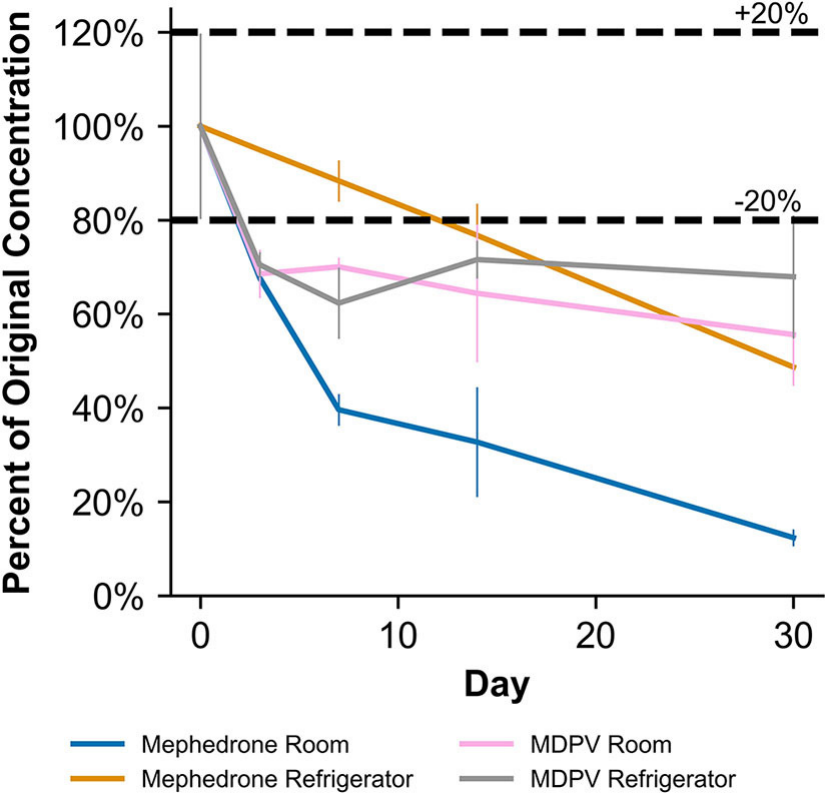
Percentage change from day 0 concentration on days 3, 7, 14, and 30 in methanolic solutions for the drugs which were deemed unstable, indicating storage conditions that contribute to instability.

**Figure 5:**
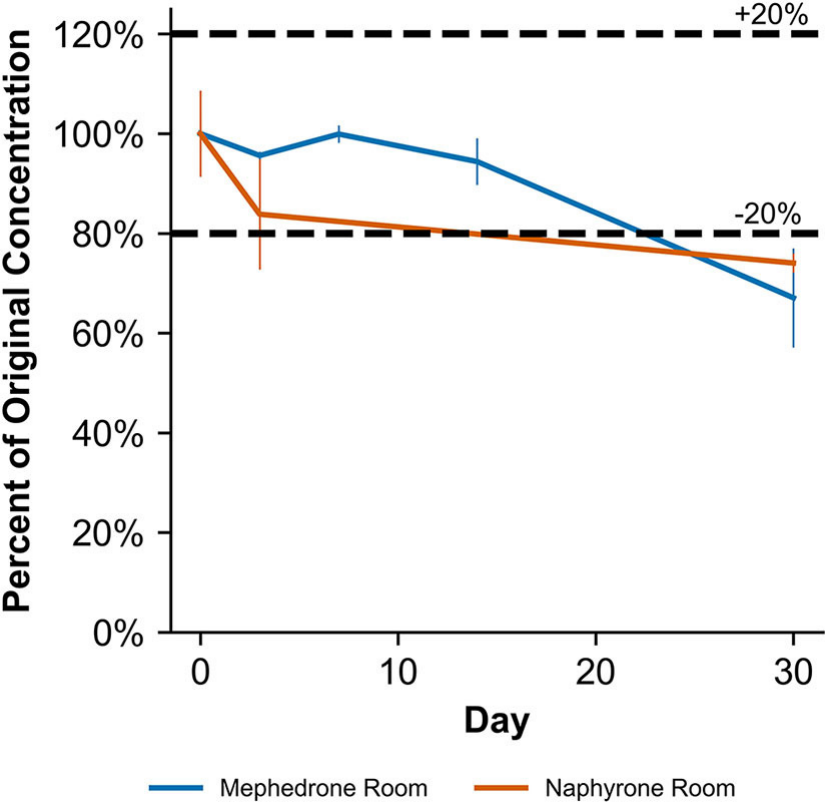
Percentage change from day 0 concentration on days 3, 7, 14, and 30 in acetonitrile solutions for the drugs which were deemed unstable, indicating storage conditions that contribute to instability.

Table 2 shows that in MeOH, mephedrone was most stable when stored in the freezer and least stable when stored at room temperature. By day 3, samples of mephedrone in MeOH stored at room temperature showed a 32.3 ± 6.1% loss of their initial concentration (Figure 4). After 30 days, 87.6 ± 3.9% of the original mephedrone concentration had degraded (Figure 4, Table 2). By comparison, mephedrone samples in MeOH stored in the refrigerator first showed a significant loss of concentration after 14 days, indicating a 23.3 ± 9.0% loss by this time (Figure 4). After 30 days, samples stored in the refrigerator showed that 51.3 ± 5.6% of the initial mephedrone concentration had degraded (Figure 4, Table 2). The most stable of these samples were those stored in the freezer, which did not demonstrate a significant loss by day 30. Notably, mephedrone was less stable in MeOH than ACN. In ACN, mephedrone was most stable when stored in the refrigerator and freezer and least stable when stored at room temperature. The ACN solutions stored at room temperature had a 32.9 ± 9.7% reduction in PAR by day 30, 10 times the amount of time than it took for MeOH samples to experience a similar loss (Figure 5, Table 2). A significant loss of PAR exceeding the method bias was not seen by the end of the study when storing mephedrone samples in ACN at refrigerator or freezer temperatures.

**Table 2:** Cathinone stability in solvents.

**Analyte**	**Solvent**	**Storage temperature (°C)**	**% Difference between days 0 and 30**	***p*-value**	**Stability call**	**First unstable day of analysis**
Mephedrone	MeOH	20	**87.6 ± 3.9%**	**3.27E-08**	**Unstable**	**Day 3**
		4	**51.3 ± 5.6%**	**1.44E-05**	**Unstable**	**Day 14**
		−20	8.7 ± 7.5%	8.41E-02	Stable	–
	ACN	20	**32.9 ± 9.7%**	**5.23E-05**	**Unstable**	**Day 30**
		4	7.2 ± 14.2%	3.77E-01	Stable	–
		−20	8.5 ± 3.3%	**5.04E-04**	Stable	–
Naphyrone	MeOH	20	**23.5 ± 30.3%**	8.84E-02	Stable	–
		4	**23.3 ± 2.7%**	1.65E-01	Stable	–
		−20	18.6 ± 4.6%	6.44E-02	Stable	–
	ACN	20	**26.0 ± 1.7%**	**1.94E-02**	**Unstable**	**Day 30**
		4	7.5 ± 3.8%	1.14E-01	Stable	–
		−20	6.6 ± 7.3%	4.80E-01	Stable	–
MDPV	MeOH	20	**44.4 ± 10.7%**	**1.84E-02**	**Unstable**	**Day 3**
		4	**32.1 ± 12.9%**	**2.51E-02**	**Unstable**	**Day 3**
		−20	**25.5 ± 9.5%**	4.80E-01	Stable	–
	ACN	20	18.6 ± 12.1%	6.79E-02	Stable	–
		4	12.1 ± 7.2%	6.70E-02	Stable	–
		−20	10.4 ± 8.7%	**1.00E-02**	Stable	–
α-PVP	MeOH	20	3.4 ± 39.7%	5.77E-01	Stable	–
		4	6.9 ± 12.2%	4.02E-01	Stable	–
		−20	2.9 ± 5.1%	**4.61E-02**	Stable	–
	ACN	20	15.1 ± 4.3%	**4.81E-06**	Stable	–
		4	7.5 ± 10.8%	**9.94E-06**	Stable	–
		−20	50.9 ± 63.5%	9.44E-01	Stable	–

*Bold values indicate instability criteria were met (i.e., % change in concentration >= 20 and/or p < 0.05)*.

**Figure 6:**
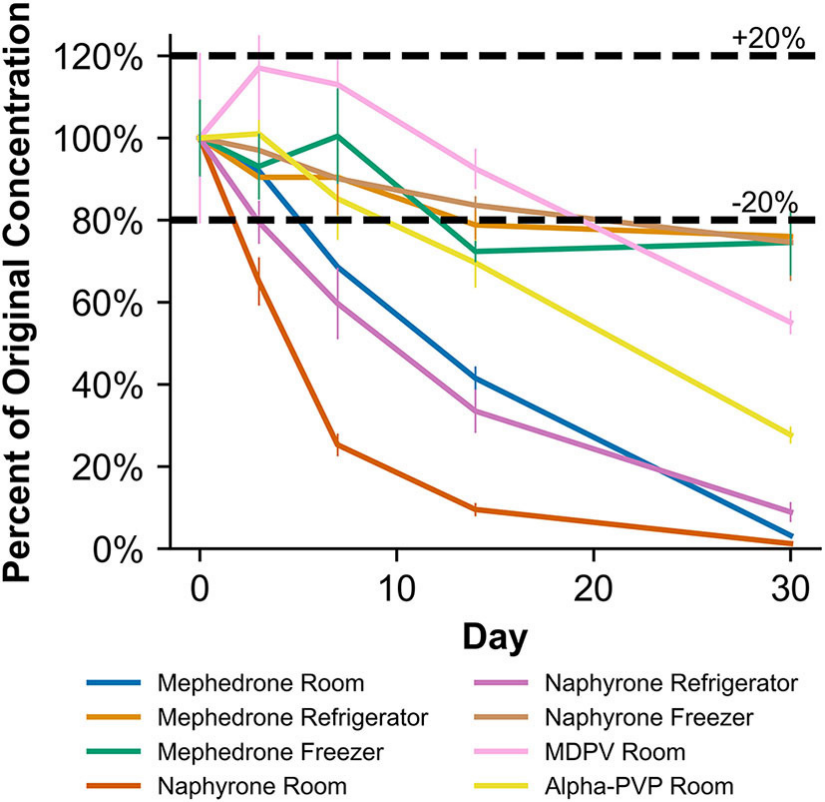
Percentage change from day 0 concentration on days 3, 7, 14, and 30 in Na_2_EDTA-preserved human whole blood samples for the drugs which were deemed unstable, indicating under storage conditions that contribute to instability.

**Figure 7:**
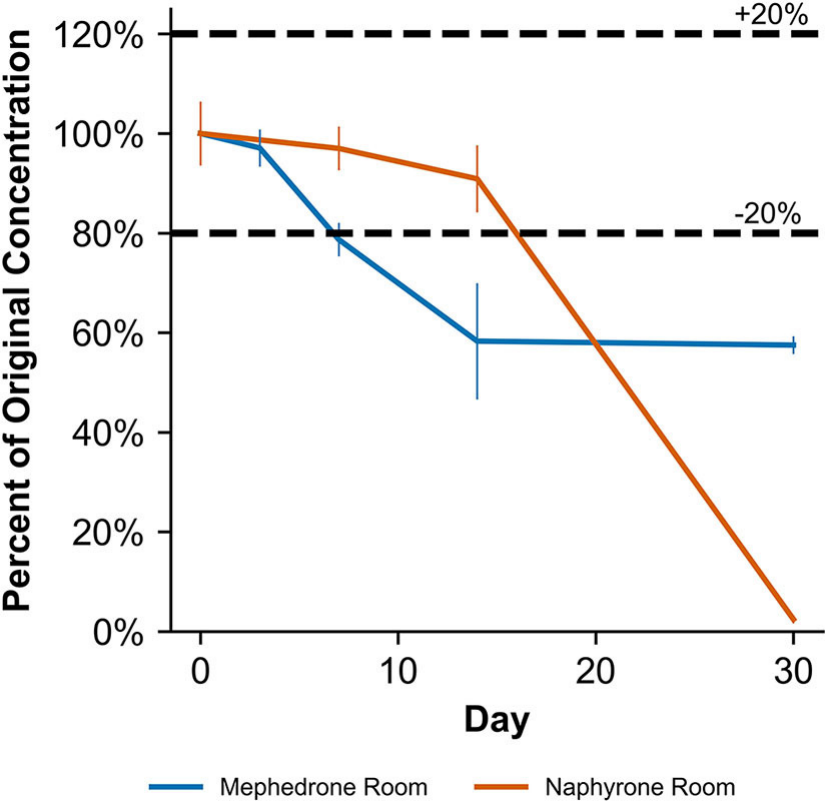
Percentage change from day 0 concentration on days 3, 7, 14, and 30 in unpreserved human urine samples for the drugs which were deemed unstable, indicating under storage conditions that contribute to instability.

When stored at room temperature, mephedrone samples in blood showed a 25.4 ± 0.7% reduction in PAR by day 7 (Figure 6). By day 30, mephedrone samples in blood showed a 96.5 ± 0.3% loss of their initial concentration (Table 3). For samples stored in the fridge, 21.2 ± 3.3% degraded after 14 days, and in the freezer, 27.6 ± 2.4% degraded during the same period (Figure 6). By day 30, samples stored at refrigerator and freezer temperatures experienced similar losses of concentration of 24.0 ± 2.1% and 25.5 ± 7.8%, respectively (Figure 6, Table 3). When stored at room temperature, mephedrone samples in urine showed a 41.7 ± 11.5% reduction after 14 days, which did not significantly change before day 30 (Figure 7, Table 3). After the duration of the study, samples stored in the refrigerator and freezer did not show a significant difference from day 0 that exceeded the method bias.

**Table 3:** Cathinone stability in biological matrices.

**Analyte**	**Solvent**	**Storage temperature (°C)**	**% Difference between days 0 and 30**	***p*-value**	**Stability call**	**First unstable day of analysis**
Mephedrone	Blood	20	**96.5 ± 0.3%**	**3.56E-09**	**Unstable**	**Day 7**
		4	**24.0 ± 2.1%**	**5.42E-03**	**Unstable**	**Day 14**
		−20	**25.5 ± 7.8%**	**3.33E-03**	**Unstable**	**Day 14**
	Urine	20	**40.8 ± 1.6%**	**5.24E-05**	**Unstable**	**Day 7**
		4	0.1 ± 5.2%	1.50E-01	Stable	–
		−20	7.0 ± 8.4%	**3.48E-02**	Stable	–
Naphyrone	Blood	20	**98.8 ± 0.2%**	**1.12E-09**	**Unstable**	**Day 3**
		4	**91.1 ± 2.2%**	**6.44E-08**	**Unstable**	**Day 3**
		−20	**25.4 ± 9.1%**	**7.65E-03**	**Unstable**	**Day 30**
	Urine	20	**97.6 ± 0.6%**	**2.44E-08**	**Unstable**	**Day 30**
		4	0.4 ± 3.1%	4.21E-01	Stable	–
		−20	2.7 ± 4.4%	2.65E-01	Stable	–
MDPV	Blood	20	**45.7 ± 3.2%**	**1.32E-03**	**Unstable**	**Day 30**
		4	4.9 ± 12.8%	3.72E-01	Stable	–
		−20	9.4 ± 3.2%	3.39E-01	Stable	–
	Urine	20	8.0 ± 5.8%	5.26E-01	Stable	–
		4	14.7 ± 8.3%	7.32E-02	Stable	–
		−20	8.6 ± 1.2%	8.23E-01	Stable	–
α-PVP	Blood	20	**72.3 ± 1.9%**	**6.75E-08**	**Unstable**	**Day 14**
		4	8.0 ± 1.2%	**9.16E-03**	Stable	–
		−20	6.6 ± 2.8%	**3.17E-03**	Stable	–
	Urine	20	12.4 ± 2.7%	**4.61E-05**	Stable	–
		4	12.4 ± 2.0%	**2.40E-02**	Stable	
		−20	14.7 ± 4.6%	**9.14E-04**	Stable	–

*Bold values indicate instability criteria were met (i.e., % change in concentration >= 20 and/or p < 0.05)*.

### Naphyrone Stability

When stored at room temperature, naphyrone samples in MeOH indicated a 23.3 ± 2.7% loss of their original concentration by day 30 (Table 2). After the same amount of time, samples stored in the refrigerator and freezer experienced no significant change in concentration by day 30 (Table 2). When stored at room temperature, naphyrone samples in ACN showed a 26.0 ± 1.7% loss of their initial concentration, indicating similar stability to storage in MeOH, unlike mephedrone. After the same amount of time, samples stored in the refrigerator and freezer remained stable.

When stored at room temperature, naphyrone samples in blood showed high instability, losing 34.9 ± 5.7% of their original concentration by day 3 and 98.8 ± 0.2% by day 30 (Figure 6, Table 3). Notably, refrigerator storage did not substantially improve naphyrone blood sample stability, still showing losses of 20.5 ± 5.1% and 91.1 ± 2.2% by days 3 and 30, respectively. Samples stored in the freezer, however, remained stable for longer, only losing 25.4 ± 9.1% over the 30-day study (Figure 6, Table 3). Similarly, when stored at room temperature, naphyrone samples in urine showed 97.6 ± 0.6% loss of their original concentration by day 30 (Figure 7, Table 3). After the same amount of time, however, samples stored in the refrigerator and freezer remained stable (Table 3).

### MDPV Stability

In MeOH, MDPV exhibited the highest stability under freezer storage temperatures and the least stability under room temperature storage temperatures (Table 2). Over the 30-day stability study, the PAR in MeOH samples stored at room temperature and in the refrigerator decreased by 44.4 ± 10.7% and 32.1 ± 12.9%, respectively. At both temperatures, samples began to show instability by day 3, losing 31.5 ± 4.9% and 29.5 ± 2.5%, respectively (Figure 4). However, at freezer temperatures, the samples showed no statistically significant loss of their initial concentration on day 0. Similarly to mephedrone, MDPV exhibited higher stability in ACN when compared to MeOH. MDPV exhibited stability when stored in ACN under room temperature, refrigerator, and freezer storage temperatures (Table 2). At the end of the 30-day stability study, none of the MDPV samples in ACN showed a statistically significant loss exceeding the method bias, regardless of their storage temperature.

In human whole blood preserved with Na_2_EDTA, MDPV exhibited the highest stability under freezer storage temperatures and the least stability under room temperature storage temperatures (Table 3, Figure 6). Over the 30-day stability study, the concentration of MDPV in human whole blood samples that were stored at room temperature decreased by 45.7 ± 3.2%. However, at refrigerator and freezer temperatures, the concentrations after 30 days remained stable. In unpreserved human urine, MDPV remained stable at all storage temperatures (Table 3).

### α-PVP Stability

α-PVP exhibited stability in both MeOH and ACN under room temperature, refrigerator, and freezer storage conditions (Table 2). In human whole blood preserved with Na_2_EDTA, α-PVP exhibited stability under refrigerator and freezer storage temperatures (Table 3) but was found to be unstable at room temperature after day 14, when 30.4 ± 5.8% was lost (Figure 6). Over the 30-day stability study, the concentration of α-PVP in whole human whole blood samples that were stored at room temperature decreased by 72.3 ± 1.9%. In urine, α-PVP was found to be stable when stored at room temperature, refrigerator, and freezer conditions.

### Impact of Chemical Structure

Overall, mephedrone was found to be the most affected by storage temperature, followed by naphyrone and then MDPV and α-PVP. Previous studies have shown that secondary amines are less stable than tertiary amines with, further substitutions such as a methylenedioxy group, present in MDPV, further increasing analyte stability (Tsujikawa et al., 2012; Glicksberg and Kerrigan, 2017). Our research has shown that a fused benzene ring reduces stability, which is a possible explanation for the difference in stability between naphyrone and α-PVP, with the additional fused benzene ring contributing to the electrophilicity of the structure. This highlights the importance of evaluating the chemical structures of these compounds in order to identify those which are likely to be least stable as they appear on the recreational drugs market.

### Implications for Forensic Toxicology

Storage temperature plays a critical role in the stability of these four synthetic cathinones in all tested matrices (MeOH, ACN, human whole blood, and human urine). This information is essential in evaluating samples in forensic casework to give accurate results to aid in court cases, particularly those in blood and urine. Human whole blood samples thought to contain mephedrone or naphyrone should be stored under freezer conditions and analyzed as quickly as possible to discourage degradation, as even at this storage temperature, there is a loss of drug over time. However, the human whole blood used in this study contained Na_2_EDTA as an anticoagulant. The long-term stability of these synthetic cathinones in blood has also been evaluated when containing sodium fluoride as a preservative and potassium oxalate as an anticoagulant (i.e., the combination in gray-top tubes) (Glicksberg and Kerrigan, 2017). However, no additional studies are published on the effects of other preservatives on the stability of this compound in blood samples. Additionally, special attention should be given when evaluating post-mortem toxicological samples suspected to contain mephedrone, naphyrone, or MDPV, particularly with regards to quantitative analysis, as before samples are collected for analysis, this blood contains neither preservatives nor anticoagulants and is at room temperature. Therefore, the stability may be affected even further, particularly in circumstances where there is a prolonged interval before sample collection.

When stored at freezer temperatures, mephedrone, naphyrone, MDPV, and α-PVP were stable in both MeOH and ACN. In the refrigerator, these compounds remained stable in ACN. However, mephedrone, naphyrone, and MDPV showed instability in MeOH under refrigerator conditions, which is a significant finding. Currently, most suppliers of forensic reference material only offer these compounds in powdered form or MeOH solutions, and most recommend refrigerator storage upon arrival in the associated material safety data sheets (MSDS) and certificates of analysis (CoA). Laboratories without the proper United States Drug Enforcement Agency (US DEA) clearance must order solvent-based 1 mg/mL solutions of Schedule I compounds rather than as powders. Although the manufacturer's recommendations may hold true for unopened amber ampules with no internal exposure to air, previous research indicates that cathinones demonstrate instability with air exposure (Tsujikawa et al., 2015). The results of this study raise cause for concern once the manufacturer-supplied ampules have been opened and exposed to air. Consequently, even laboratories that follow the recommendations of the manufacturer from which they order their standards could experience detrimental effects from this instability when determining quantitative values. This is particularly relevant for substances such as synthetic cathinones, which may not be part of routine analysis, increasing the time period reference material may be in circulation within the laboratory.

The MSDS and CoA associated with cathinone standard solutions also often recommend storage in a dark location. This recommendation indicates that uncontrolled light exposure could also contribute to cathinone instability in solvents. The results presented here were acquired under uncontrolled light conditions, which may partially account for the high standard deviations sometimes associated with solvent instability over the 30 days. Although these high standard deviations did not show statistical significance indicating instability in all cases, minimizing the effects of light/dark cycles is expected to improve future analytical data. Making diluted working solutions in ACN rather than MeOH and storing them in amber vials under freezer conditions rather than the refrigerator when laboratories receive these standards may be beneficial. Taking these precautionary steps can help to safeguard against reporting concentrations that are artificially raised by degrading standard solutions used for generating calibration curves and positive controls.

### Future Work

Although this paper examined the stability of mephedrone, naphyrone, MDPV, and α-PVP in MeOH, ACN, human whole blood, and human urine it did so only for a period of 30-days. Due to the continual identification of new NPSs, it may be that retrospective analysis in samples for these analytes does not happen within a 30-day window. As a result, further work is needed to establish the long-term stability of these analytes within these matrices and solvents. This study did also not investigate the impact of freeze/thaw cycles on biological samples as this parameter is investigated during method development. Although reference material typically does not freeze, cold/warm cycles still occur when this material is removed from the fridge or freezer. This impact of this parameter should, therefore, be investigated when dealing with reference material in future work. Finally, it has been shown that pH also plays a significant role in analyte stability, particularly with urine samples. However, this parameter is being investigated as part of a larger study.

## Conclusions

Understanding the stability of reference material is fundamental within the field of forensic toxicology in order to ensure accurate and reliable results are reported. The use of unstable reference material can lead to the artificial inflation of results, which could have a significant impact upon the justice system and the individuals whose samples are being tested in casework. The stability of four synthetic cathinones, mephedrone, naphyrone, MDPV, and α-PVP, were assessed over a 30-day period when stored in MeOH, ACN, human whole blood, and human urine, at room, fridge, and freezer temperatures.

Overall, increased stability was shown when samples were stored in ACN, as opposed to MeOH. Mephedrone was shown to be the least stable of the four cathinones monitored and α-PVP the most. Samples showed the largest level of degradation when stored at room temperature and showed the greatest stability when stored in the freezer.

Although unopened reference material may be stable for a long period of time, this work highlights the importance of renewing reference material on a regular basis once opened.

When introducing new NPS drugs into the laboratory, analysts and reporting officers should be aware that these compounds may not be stable, and care should be taken when interpreting results. Where possible, extended stability studies should be carried out during validation to evaluate the stability of these compounds to determine the potential impact that instability may have upon casework.

## Data Availability Statement

The original contributions presented in the study are included in the article/supplementary materials, further inquiries can be directed to the corresponding author.

## Author Contributions

LN and KS contributed to the conception and design of the study. HC and LR acquired and analyzed experimental data. HC, LN, and KS drafted the manuscript. All authors were involved in the interpretation of experimental data and manuscript revision. All authors contributed to the article and approved the submitted version.

## Conflict of Interest

The authors declare that the research was conducted in the absence of any commercial or financial relationships that could be construed as a potential conflict of interest.
